# Performance assessment of a duplex quantitative PCR assay for detecting *Leishmania infantum* and *Leishmania tarentolae* using three qPCR devices

**DOI:** 10.1128/spectrum.03854-25

**Published:** 2026-06-15

**Authors:** Maria Stefania Latrofa, Lucas Cafferati-Beltrame, Pietro D'Addabbo, Marco Cereda, Viviane Noll Louzada-Flores, Ilaria Varotto Boccazzi, Sara Epis, Jairo Alfonso Mendoza-Roldan, Domenico Otranto

**Affiliations:** 1 Department of Veterinary Medicine, University of Bari 66177, Bari, Italy; 2 Department of Biosciences, Biotechnologies and Environment, University of Bari 837102, Bari, Italy; 3 STMicroelectronics Srl 68232 , Agrate Brianza, Italy; 4 Unité de Parasitologie Moléculaire et Signalisation, Institut Pasteur, Université Paris Cité, INSERM 1201 555089, Paris, France; 5 Department of Biosciences, University of Milan 98853, Milan, Italy; 6 Department of Veterinary Clinical Sciences, City University of Hong Kong 53025, Kowloon Tong, Hong Kong, China; Institut de recherche pour le developpement 98751 , Montpellier, France

**Keywords:** droplet digital PCR, real-time PCR, point-of-care, lab-on-chip, vector-borne disease, *Leishmania tarentolae*, *Leishmania infantum*

## Abstract

Among *Leishmania* spp. transmitted by phlebotomine sand flies, *Leishmania infantum* is regarded as the most widely distributed species worldwide and the primary causative agent of canine leishmaniasis. However, in regions where *L. Infantum* is endemic the sympatric occurrence of the herpetophilic *Leishmania tarentolae* in dogs, cats, and humans has prompted the development of sensitive and specific diagnostic assays. To evaluate the clinical and analytical sensitivities of a quantitative PCR (qPCR) assay targeting the minicircle kDNA fragment of both species, three devices with different technologies (lab-on-chip real-time PCR applied on the portable Q3-Plus V2 platform, CFX96 Real-Time System, and droplet digital PCR [ddPCR]) were tested. The sensitivity (limit of detection [LOD]) and specificity of the assay were assessed by processing DNA samples from cultures of both *Leishmania* spp. and from biological samples of *Sergentomyia minuta* (*n* = 120; midgut), of lizards (*Podarcis siculus*; *n* = 29; blood, eggs, heart, intestine, kidneys, liver, lungs, and spleen), and of dogs (*n* = 101; blood, bone marrow, conjunctival swab, lymph node, and skin). The performance of the assay applied on all devices was evaluated by comparing the quantification cycle values obtained by qPCR with the DNA copy number determined by ddPCR. The LOD of serial dilutions of DNA from each *Leishmania* spp., spiked DNA, and biological samples were overlapping across the devices, whereas receiver operating characteristic analysis showed a shift in sensitivity for ddPCR (AUC, 0.9858) compared to CFX96 and Q3 qPCRs (AUC, 0.9517). The assay developed is an efficient and sensitive tool regardless of the device tested, though ddPCR showed higher sensitivity in detecting low quantities of DNA in all samples, as supported by the highest AUC value.

**IMPORTANCE:**

The development of sensitive and specific molecular tools for detecting *Leishmania infantum* and *Leishmania tarentolae* has been evaluated in a duplex qPCR assay applied on three devices with different technologies (lab-on-chip real-time PCR applied on the portable Q3-Plus V2 platform, CFX96 Real-Time System, and droplet digital PCR). Both *Leishmania* spp. were tested in different tissues of various animal hosts, and the clinical sensitivity and specificity were evaluated. The duplex qPCR assay developed herein proved to be an efficient tool, regardless of the device used, and will be useful in improving current knowledge on the distribution of *L. infantum* and of *L. tarentolae* and in better understanding the potential role of new hosts/reservoirs or vectors for these *Leishmania* spp.

## INTRODUCTION

Among vector-borne diseases endemic in tropical and subtropical regions (1), the leishmaniases caused by *Leishmania* spp. (Trypanosomatida: Trypanosomatidae) represent a risk for 350 million people in endemic regions and infect 12 million people (2). *Leishmania infantum* is the primary causative agent of canine leishmaniasis (CanL), being a zoonotic species widely distributed throughout South and Central America, Southwest Asia, and the Mediterranean basin (3, 4). In the last region, *L. infantum* may occur in sympatry with other pathogenic species, such as *Leishmania tropica*, *Leishmania major,* and *Leishmania donovani* (5–7). In recent years, *Leishmania tarentolae* (subgenus *Sauroleishmania*) has also been detected (8), predominantly in reptiles (e.g., *Podarci*s *siculus* lizards and *Tarentola mauritanica* geckos), and is transmitted by the herpetophilic sand fly *Sergentomyia minuta* (Diptera: Psychodidae) (9, 10). This non-pathogenic species of *Leishmania* has been detected serologically and molecularly in humans, dogs, and cats (8, 11–13). The competence of *L. tarentolae* to infect mammalian hosts was further supported by experimental studies in which this protozoon successfully colonized, survived, and persisted in primary canine monocyte-derived mononuclear cells (14), as well as in experimentally infected dogs (15). The abovementioned evidence shed light on the interpretation of the results of diagnostic serological tests, as both *L. infantum* and *L. tarentolae* may occur simultaneously in clinically healthy dogs (12). The latter also suggests a role for *L. tarentolae* in producing pro-inflammatory cytokines and exerting cross-immune protection against CanL by *L. infantum* (8, 16). To better understand the epidemiology of *L. tarentolae*, sensitive molecular protocols based on quantitative PCR (qPCR) technologies have been developed (17, 18). In addition, the market demand for increasingly sensitive, innovative, and affordable point-of-care diagnostic devices has been evaluated for the detection of *L. infantum* infection. For example, the lab-on-chip qPCR applied on the portable Q3-Plus V2 platform (Q3 qPCR) showed higher performance than the Bio-Rad CFX96 Real-Time System (CFX96 qPCR) in detecting *L. infantum* DNA from non-extracted dog samples (19). Similarly, a droplet digital PCR (ddPCR) method has been developed for detecting *Leishmania* spp. (18, 20, 21).

In this study, a duplex qPCR assay targeting the minicircle kDNA of *L. infantum* and *L. tarentolae* was developed*,* and its analytical and clinical sensitivities were evaluated using Q3 qPCR, ddPCR, and conventional CFX96 qPCR by testing different host and tissue samples.

## MATERIALS AND METHODS

### DNA samples

Genomic DNA (gDNA) of reference *Leishmania* strains was obtained from cultures of *L. infantum* (zymodeme MON-1 MHOM/TN/80/IPT1), *L. tarentolae* (RTAR/IT/81/ISS21-G.6c/LEM-124), *L. major* (WR2885), and *L. tropica* (L747), extracted using the QIAamp DNA Micro Kit (Qiagen GmbH, Hilden, Germany) following the manufacturer’s instructions. The gDNA from 250 biological samples divided among lizard (*n* = 29; blood, eggs, heart, intestine, kidneys, liver, lungs, spleen), *Se. minuta* (*n* = 120; midgut) collected from a previous study [8], and dogs naturally infected with *L. infantum* (*n* = 92; blood, bone marrow, conjunctival swab, lymph node, and skin) and experimentally infected with *L. tarentolae* (*n* = 9; bone marrow, conjunctival swab, and lymph node) was included in the study (Table 1). In addition, gDNA of biological samples (*n* = 12 lizards; heart, intestine, kidneys; *n* = 20 dogs; bone marrow, lymph node, and skin) previously tested negative for *Leishmania* spp. was used as negative controls.

**TABLE 1:** Quantification cycle (Cq), DNA concentration (ng/μL), and copy number (copies/20 µL reaction) values obtained from *Leishmania infantum-* and *Leishmania tarentolae*-positive samples using the CFX96 qPCR, Q3 qPCR, and ddPCR

Biological samples	*Leishmania infantum*					
	Dog					
	CFX96 qPCR		Q3 qPCR		ddPCR	
	Cq Min-Mean-Max	ng/μl Min-Mean-Max	Cq Min-Mean-Max	ng/μl Min-Mean-Max	Copies/20 µl Min-Mean-Max	ng/μl Min-Mean-Max
Bone marrow (*n* = 20)	17.64-29.78-35.64	4.33 × 10^−7^-5.19 ×10^−3^-9.88 × 10^−2^	17.44-29.60-37.54	7.33 × 10^−9^-2.67 × 10^−4^-4.96 × 10^−3^	5.65-14,302.38-245,051.37	3.84 × 10^−7^-5.03 × 10^−4^-8.35 × 10^−3^
(*n* = 4)^*a*^	neg	neg	neg	neg	neg	neg
Lymph node (*n* = 21)	18.59-28.48-36.19	2.97 × 10^−7^-6.13 × 10^−3^-5.15 × 10^−2^	19.02-28.74-36.74	1.25 × 10^−8^-2.52 × 10^−4^-1.72 × 10^−3^	5.69-15,524.72-85,076.96	3.86 × 10^−7^-5.91 × 10^−4^-3.10 × 10^−3^
(*n* = 2)^*a*^	neg	neg	neg	neg	neg	neg
Blood (*n* = 9)	29.3-34.21-37.89	9.25 × 10^−8^-7.99 × 10^−6^-3.34 × 10^−5^	30.30-34.08-37.89	5.82 × 10^−9^-2.35 × 10^−7^-9.20 × 10^−7^	4.62-95.49-479.25	3.18 × 10^−7^-5.08 × 10^−6^-2.44 × 10^−5^
Skin (*n* = 24)	24.13-33.60-37.58	1.14 × 10^−7^-6.92 × 10^−5^-1.16 × 10^−3^	26.90-34.27-37.68	6.67 × 10^−9^-6.19 × 10^−7^-8.91 × 10^−6^	4.29-521.75-6,560-40	2.96 × 10^−7^-2.33 × 10^−5^-2.82 × 10^−4^
Conjunctival swab (*n* = 18)	25.56-32.01-37.21	1.48 × 10^−7^-4.13 × 10^−5^-4.34 × 10^−4^	23.20-31.74-37.81	6.14 × 10^−9^-6.67 × 10^−6^-1.05 × 10^−4^	4.35-779.27-8,988	3.00 × 10^−7^-3.46 × 10^−5^-3.79 × 10^−4^
(*n* = 3)^*a*^	neg	neg	neg	neg	neg	neg
Total (*n* = 101)						

Sand fly						
Midgut (*n* = 120)	29.83-35.80-37.97	8.76 × 10^−8^-1.53 × 10^−6^-2.32 × 10^−5^	27.135-35.268-38.071	5.15 × 10^−9^-2.80 × 10^−7^-7.64 × 10^−6^	4.09-1,004.32-28,557.34	2.83 × 10^−7^-4.18 × 10^−5^-1.12 × 10^−3^
						

Biological samples	*Leishmania tarentolae*					
	Dog					
	CFX96 qPCR		Q3 qPCR		ddPCR	
	Cq Min-Mean-Max	ng/μl Min-Mean-Max	Cq Min-Mean-Max	ng/μl Min-Mean-Max	Copies/20 µl Min-Mean-Max	ng/μl Min-Mean-Max
Bone marrow (*n* = 20)	neg	neg	neg	neg	4.51-11.63-18.76	8.91 × 10^−7^-2.12 × 10^−6^-3.36 × 10^−6^
(*n* = 4)^*a*^	33.47-35.24-38.23	1.69 × 10^−6^-2.30 × 10^−5^-4.61 × 10^−5^	31.93-36.19-38.83	1.12 × 10^−7^-3.09 × 10^−5^-1.11 × 10^−6^	10.84-98.37-210	2.02 × 10^−6^-1.54 × 10^−5^-3.18 × 10^−5^
Lymph node (*n* = 21)	neg	neg	neg	neg	4.27-27.55-50.83	8.47 × 10^−7^-4.67 × 10^−6^-8.50 × 10^−6^
(*n* = 2)^*a*^	33.33-34.85-36.38	6.10 × 10^−6^-2.84 × 10^−5^-5.08 × 10^−5^	32.73-35.12-37.51	2.70 × 10^−7^-3.37 × 10^−6^-6.47 × 10^−6^	32.18-102.09-172	5.55 × 10^−6^-1.60 × 10^−5^-2.64 × 10^−5^
Blood (*n* = 9)	neg	neg	neg	neg	neg	neg
Skin (*n* = 24)	neg	neg	neg	neg	10-13-16	1.87 × 10^−6^-2.38 × 10^−6^-2.90 × 10^−6^
Conjunctival swab (*n* = 18)	neg	neg	neg	neg	9.37	1.76 × 10^−6^
(*n* = 3)^*a*^	34.24-35.63-37.02	3.91 × 10^−6^-1.54 × 10^−5^-2.70 × 10^−5^	36.02	7.27 × 10^−7^	4.83-45.31-120.71	9.50 × 10^−7^-7.30 × 10^−6^-1.90 × 10^−5^
Total (*n* = 101)						

Lizard						
Lungs (*n* = 5)	28.25-32.14-34.84	1.78 × 10^−5^-4.39 × 10^−4^-1.73 × 10^−3^	28.03-31.85-35.49	1.03 × 10^−6^-4.43 × 10^−5^-1.48 × 10^−4^	230.23-484.81-1,170.91	3.47 × 10^−5^-6.84 × 10^−5^-1.58 × 10^−4^
Spleen (*n* = 6)	29.27-32.84-36.19	6.96 × 10^−6^-2.74 × 10^−4^-8.52 × 10^−4^	31.05-33.47-36.34	5.86 × 10^−7^-8.90 × 10^−6^-1.98 × 10^−5^	27.09-477.74-1,657.57	4.73 × 10^−6^-6.55 × 10^−5^-2.18 × 10^−4^
Liver (*n* = 4)	26.03-32.15-36.14	7.21 × 10^−6^-2.71 × 10^−3^-8.09 × 10^−3^	33.64-34.72-36.33	5.90 × 10^−7^-2.20 × 10^−6^-3.54 × 10^−6^	10.18-8,223.64-32,482.6	1.90 × 10^−6^-8.85 × 10^−4^-3.48 × 10^−3^
Heart (*n* = 5)	30.77-32.08-32.88	6.94 × 10^−5^-1.51 × 10^−4^-3.00 × 10^−4^	30.06-31.13-31.82	1.19 × 10^−5^-2.16 × 10^−5^-3.83 × 10^-5^	35.38-195.14-432.77	6.06 × 10^−6^-2.91 × 10^−5^-6.24 × 10^−5^
Intestine (*n* = 1)	29.88	5.84 × 10^−4^	30.73	2.46 × 10^−5^	508.01	7.25 × 10^−5^
Kidneys (*n* = 3)	35.85	8.82 × 10^−6^	neg	neg	7.201-29.92-53.48	1.38 × 10^−6^-5.11 × 10^−6^-8.91 × 10^−6^
Eggs (*n* = 2)	29.34-29.35-29.36	8.00 × 10^−4^-8.06 × 10^−4^-8.11 × 10^−4^	29.21-29.99-30.77	2.40 × 10^−5^-4.57 × 10^−5^-6.74 × 10^−5^	1,224.1-1,229.6-1,235.1	1.64 × 10^−4^1.65 × 10^−4^-1.66 × 10^−4^
Blood (*n* = 3)	25.85-27.93-31.88	1.39 × 10^−4^-5.76 × 10^−3^-9.16 × 10^−3^	23.96-27.88-30.74	2.44 × 10^−5^-7.76 × 10^−4^-2.22 × 10^−3^	851.3-54,543.72-156,264.81	1.17 × 10^−4^-5.31 × 10^−3^-1.50 × 10^−2^
Total (*n* = 29)						

Sand fly						
Midgut (*n* = 120)	15.31-27.73-36.91	4.22 × 10^−6^-7.58 × 10^−1^-1.39 × 10^+1^	14.442-28.370-38.657	1.26 × 10^−7^-3.48 × 10^−2^-1.25	4.614-28,617,862.44−2.0 × 10^8^	9.10 × 10^−7^-1.68-11.7

^*a*^ Dogs experimentally infected with *L. tarentolae*; Minimum, mean, and maximum of Cq values are indicated.

### Primers and probe for *Leishmania infantum* and *Leishmania tarentolae* detection

Primers for *Leishmania* spp. (*Leish*.spp_k_F: 5′-TATTTTACACCAACCCC-3′/*Leish*.spp_k_R: 5′-GTAGGGGCGTTCTGC-3′) and a TaqMan-MGB hydrolysis probe (VIC-5′-CACACGAAAATTCG-3′-non-fluorescent quencher-MGB; Applied Biosystems; Foster City, CA, USA) targeting a partial fragment of kDNA of *L. tarentolae* (120 bp) were designed in this study by alignment of sequences from representative *Leishmania* spp. (*L. infantum* GenBank accession: EU437403; *L. tarentolae* GenBank accession: K01980; *L. major* GenBank accession: U51719; *L. tropica* GenBank accession: MH511158) using Primer Express 2.0 (Applied Biosystems, Foster City, CA). The specificity of the probe for *L. tarentolae* was confirmed *in silico* using the Basic Local Alignment Search Tool (BLAST, GenBank, NCBI) and by testing gDNA from the above *Leishmania* spp. *Leishmania infantum* DNA was detected using primers (*Leish*-1, 5′-AACTTTTCTGGTCCTCCGGGTAG-3′ and *Leish*-2, 5′-ACCCCCAGTTTCCCGCC-3′) and a TaqMan-MGB hydrolysis probe (FAM-5′-AAAAATGGGTGCAGAAAT-3′-non-fluorescent quencher-MGB) targeting kDNA (124 bp) and the protocol previously described (22).

### CFX96 qPCR protocol

gDNA samples were tested with the CFX96 qPCR (Bio-Rad Laboratories, Inc., Hercules, CA, USA) using the primers and hydrolysis probes described above. The 20 μL reaction mixture contained 10 μL of SsoAdvanced Universal Probes Supermix (Bio-Rad Laboratories, Hercules, CA, USA), 6.68 μL of diethyl pyrocarbonate (DEPC) treated pyrogen-free DNase/RNase-free water (Invitrogen, Carlsbad, CA, USA), primers (900 nM each), hydrolysis probes (200 nM for VIC and 300 nM for FAM), and 1.6 μL of DNA sample. The run protocol (duration, 1 h and 13 min) consisted of a hot start at 95°C for 3 min followed by 50 cycles of denaturation (95°C for 10 s) and annealing-extension (60°C for 30 s). All samples were tested in duplicate, and DNA from negative and positive controls, as well as a no-template control, was included in each qPCR run. The increase in the fluorescent signal was recorded during the extension step of the reaction, and the data analyzed using CFX Manager Software Maestro (Bio-Rad Laboratories, Inc., Hercules, CA, USA).

### Q3 qPCR protocol

gDNA samples were tested with the Q3-Plus V2 (STMicroelectronics S.r.l., Italy), a compact platform based on a rapid and easy-to-use real-time PCR method and a disposable cartridge, as previously described (19). The Q3 qPCR reaction mixture consisted of a 5 μL volume containing 2.5 μL of SsoAdvanced Universal Probes Supermix (Bio-Rad Laboratories, Hercules, CA, USA), 1.52 μL of DEPC water, primers and hydrolysis probes at the same concentrations used in the CFX96 qPCR, and 0.6 μL of DNA sample. The run protocol (duration, 1 h and 26 min) consisted of a wax-melting step at 95°C for 100 s, a hot start step at 95°C for 200 s, followed by 50 cycles of denaturation at 95°C for 10 s, annealing-extension at 58°C for 15 s, and fluorescence acquisition at 60°C for 15 s with the following optical parameters: exposure time, 1 s; gain, 16; and LED power, 7 for VIC, and exposure time, 1 s; gain, 15; and LED power, 6 for FAM.

### Droplet digital PCR protocol

gDNA samples were tested in duplicate with the QX200 instrument (Bio-Rad Laboratories, Inc., Hercules, CA, USA) using the primers and hydrolysis probes described above. The 20 μL reaction mixture contained 11 μL of digital PCR Supermix for probes (no dUTP) (Bio-Rad Laboratories, Hercules, CA, USA), 5.68 μL of DEPC-treated pyrogen-free DNase/RNase-free water (Invitrogen, Carlsbad, CA, USA), primers and hydrolysis probes at the same concentrations used in the CFX96 and Q3 qPCRs, and 1.6 μL of DNA sample. Each reaction was then loaded into a sample well of a Bio-Rad DG8 droplet generator cartridge, along with 70 μL of droplet generation oil. Droplets were formed using QX200 droplet generators and then transferred to a 96-well PCR plate to perform PCR (T100 Thermal Cycler, Bio-Rad Laboratories, Inc., Hercules, CA, USA). Thermal-cycling conditions (duration: 3 h and 15 min) consisted of a hot start at 95°C for 10 min with a 2°C/s ramp rate, followed by 50 cycles of denaturation at 95°C for 30 s with a 2°C/s ramp rate and annealing-extension at 52°C for 90 s with a 2°C/s ramp rate, followed by 98°C for 10 min and 4°C for 30 min. The products were registered on a QX200 Droplet Reader and analyzed using QuantaSoft software (version 1.7.4, Bio-Rad Laboratories), and results expressed as DNA copy number per 20 μL (copies/20μL) of reaction. For wells flagged as “No Call,” visual inspection and manual setting of the threshold value were performed. DNA from positive and negative controls was included in each ddPCR run to remove the background signals.

### Analytical sensitivity (limit of detection), specificity, and efficiency

The limit of detection (LOD), amplification efficiency (E), correlation coefficient (R^2^), and slope obtained by Q3 and CFX96 qPCRs were calculated based on a standard curve constructed using 10-fold dilution series of the DNA from promastigotes of *L. infantum* (concentration from 8.55 × 10^−4^ ng/μL to 8.55 × 10^−8^ ng/µL), *L. tarentolae* (concentration from 1.154 × 10^−3^ ng/µL to 1.154 × 10^−7^ ng/µL) and the spiked DNA from both *Leishmania* spp. (*L. infantum* concentration from 4.27 × 10^−3^ ng/µL to 4.27 × 10^−10^ ng/µL and for *L. tarentolae* from 5.77 × 10^−3^ ng/µL to 5.77 × 10^−10^ ng/µL). Each dilution was tested in triplicate in the CFX96 qPCR and in duplicate in the Q3 qPCR. The amount of purified DNA from each *Leishmania* strain was determined using NanoDrop One^C^ instrument (Thermo Fisher Scientific, Waltham, MA, USA).

The LOD, R^2^, and E obtained by ddPCR were calculated using the same 10-fold dilution series of DNA from *Leishmania* spp. cultures tested in CFX96 and Q3 qPCRs. Samples were considered positive (blue and green droplets for FAM and VIC, respectively) if more than three fluorescent signal events were observed above the threshold line. The cut-off was defined based on negative samples showing no more than two fluorescent signal events.

The performance of all devices in detecting *Leishmania* spp. DNA was assessed by converting ddPCR DNA copy numbers to ng/μL and Cq values and comparing with the values obtained in the Q3 and CFX96 qPCRs. The clinical sensitivity and ability of the assay to detect *Leishmania* spp. DNA in biological samples was assessed by testing 10-fold dilution series of DNA from representative positive samples, one for each tissue, having the highest parasite burden expressed in Cq value (Table 3).

### Statistical analysis

Boxplots analyses were generated with GraphPad Prism v8.4.2 (GraphPad Software, San Diego, CA, USA) for exploratory visualization. The plots displayed the Cq values for all positive samples tested by the CFX96 and Q3 qPCRs and copies/20 µL for the ddPCR results. The diagnostic sensitivity (Se) and specificity (Sp) of the assay for testing biological samples using the CFX96, Q3 qPCR, and ddPCR were compared by plotting the receiver operating characteristic (ROC) curves (plots of sensitivity against [1 − specificity]) for each fluorophore. The area under the ROC curve (AUC) was estimated by non-parametric integration (23) to measure diagnostic accuracy. ROC analyses were performed using RStudio version 4.5.0.

## RESULTS

A duplex qPCR assay was designed for the detection of *L. infantum* and *L. tarentolae* in different host and tissue samples. The analytical and clinical sensitivities of the assay were evaluated on three devices (CFX96 qPCR, Q3 qPCR, and ddPCR), each having a different technology.

**Fig 1:**
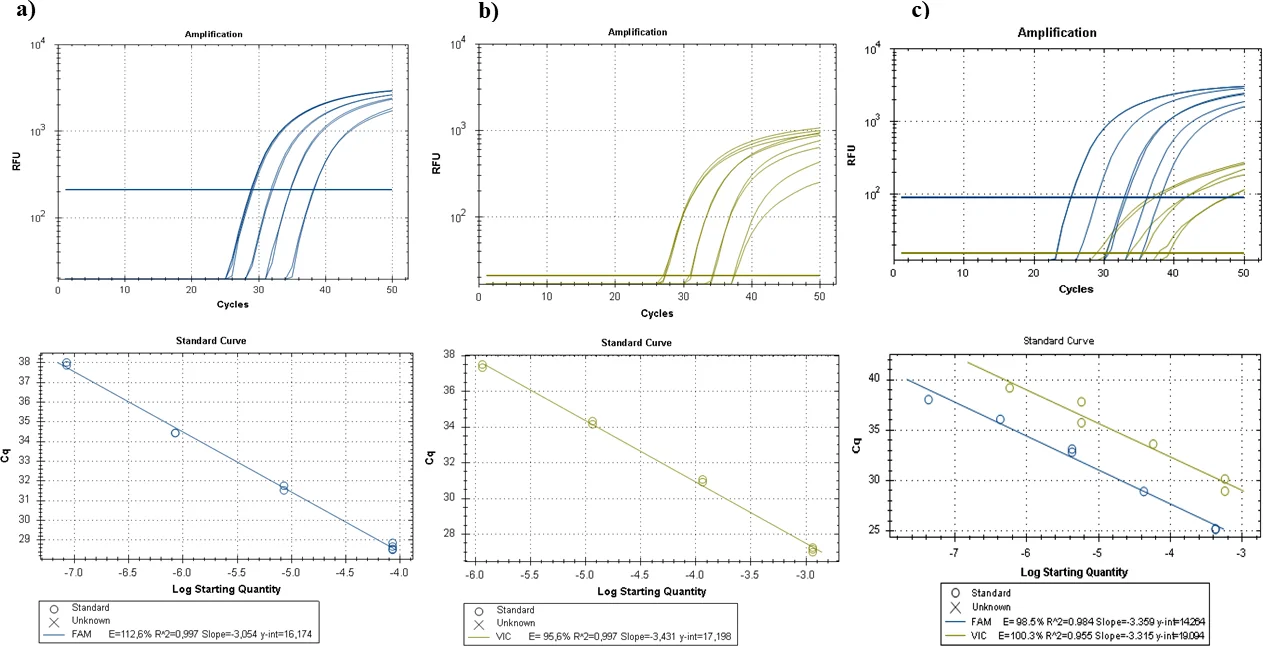
Standard curves of *Leishmania* spp. DNA obtained by the CFX96 qPCR platform. The amplification plots represented by the fluorescent signal, expressed in relative fluorescence units (RFU), and standard curves generated from serial dilutions of genomic DNA from *L. infantum* (**a**), *L. tarentolae* (**b**), and spiked DNA from *L. infantum* and *L. tarentolae* (**c**).

**Fig 2:**
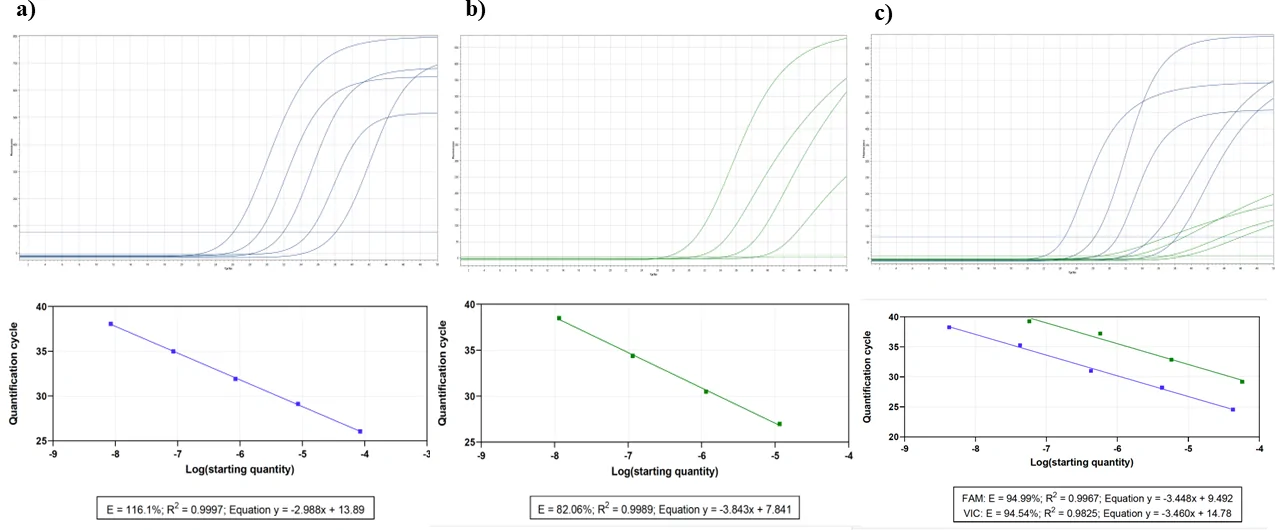
Standard curves of *Leishmania* spp. DNA obtained by Q3 qPCR platform. The amplification plot represented by the fluorescent signal, expressed in relative fluorescence units (RFU), and standard curves generated from serial dilutions of genomic DNA from *L. infantum* (**a**), *L. tarentolae* (**b**), and spiked DNA from *L. infantum* and *L. tarentolae* (**c**).

**Fig 3:**
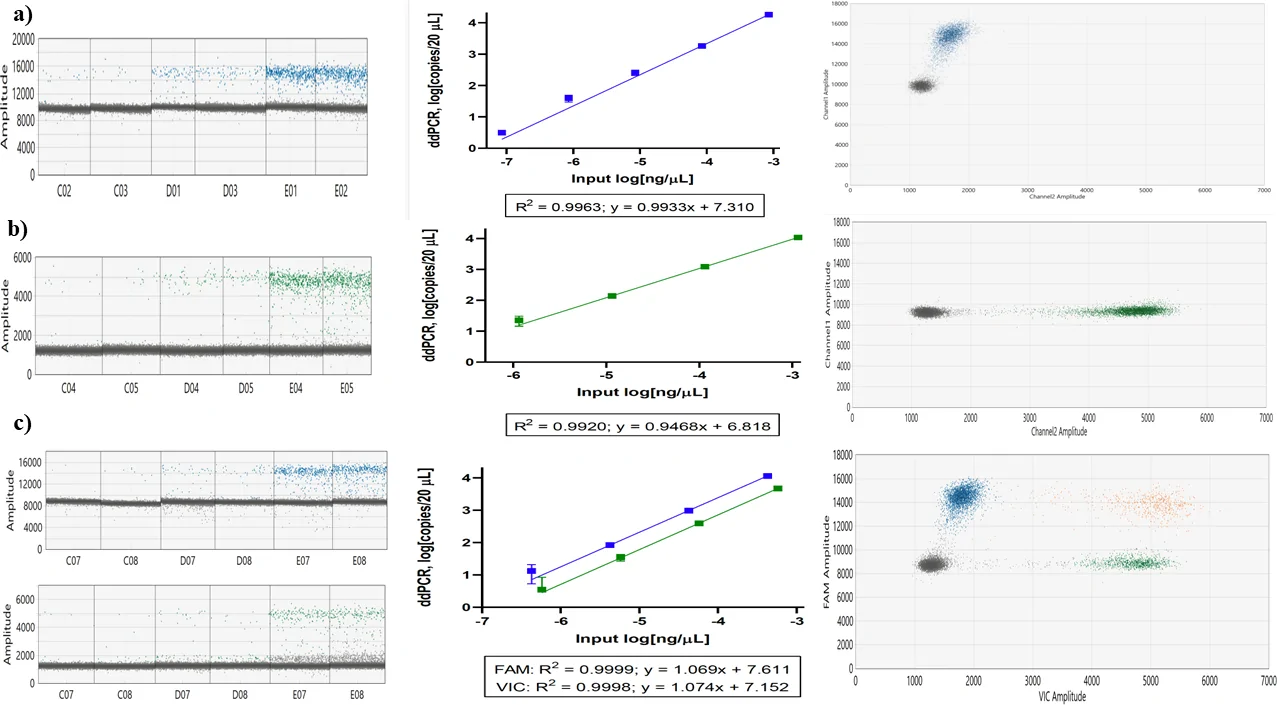
Standard curves of *Leishmania* spp. DNA obtained by ddPCR platform. The amplification plots represented by the fluorescent signal according to the amplitude, droplet events (1D and 2D fluorescence plots), and DNA concentration (ng/μL) obtained from serial dilutions of *L. infantum* DNA (**a**, blue droplets); *L. tarentolae* DNA (**b**, green droplets); and spiked DNA from *L. infantum* and *L. tarentolae* (**c**, droplets positive for both species are represented in orange). The negative-control samples are represented by black droplets.

A single positive fluorescent signal was detected in CFX96 and Q3 qPCRs, and ddPCR for each culture of *L. infantum* and *L. tarentolae*, whereas two species-specific fluorescent signals were detected for the spiked DNA of both species and for the co-infected positive biological samples. The optimization of the assay in both CFX96 and Q3 qPCRs was confirmed by the value of E (from 95.6% to 112.6% and 82.06% to 116.1% for the CFX96 and Q3 qPCRs), R^2^ (from 0.955 to 0.997 and 0.9825 to 0.9997 for the CFX96 and Q3 qPCRs), and slope (from −3.054 to −3.431 and −2.988 to −3.843 for the CFX96 and Q3 qPCRs) obtained from the standard curves (Table 2; Fig. 1 and 2). Meanwhile, ddPCR showed a robust linear relationship between the input DNA concentration and the calculated copy number per 20 μL reaction (R^2^, from 0.9963 to 0.9999 for *L. infantum*, *L. tarentolae,* and for the spiked DNA) for each *Leishmania* spp. and for the spiked DNA samples (Table 2; Fig. 3). The LOD recorded in CFX96 qPCR was up to 8.55 × 10^−7^ ng/µL for *L. infantum* isolates (Cq, 37.9), 1.154 × 10^−6^ ng/µL for *L. tarentolae* (Cq, 37.6), and up to 4.27 × 10^−7^ ng/µL for *L. infantum* (Cq, 38.03) and 5.77 × 10^−6^ ng/µL for *L. tarentolae* (Cq, 39.2) for spiked DNA samples. The LOD of the CFX96 qPCR overlapped those obtained in the tQ3 qPCR (Fig. 1 and 2). The LOD for the ddPCR was of 3.13 ± 0.516 copies/20 μL (1.38 × 10^−7^ ng/µL, Cq 38.29) for *L. infantum*, 22.5 ± 7.99 copies/20 μL for *L. tarentolae* (1.69 × 10^−6^ ng/µL, Cq, 37.45), and for the spiked DNA of 13.24 ± 7.863 copies/20 μL (8.51 × 10^−7^ ng/µL, Cq 35.98) and 3.52 ± 4.978 copies/20 μL (7.07 × 10^−7^ ng/µL, Cq 39.15) for *L. infantum* and *L. tarentolae*, respectively (Fig. 3).

**TABLE 2:** Equation of standard curves, R^2^ values, and amplification efficiency generated by CFX96 qPCR, Q3 qPCR, and ddPCR according to *Leishmania infantum*, *Leishmania tarentolae,* and the spiked DNA of both *Leishmania* spp.

	Slope	Y-intercept	R^2^	Efficiency (%)
CFX96 qPCR				
*L. infantum*	−3.054	y-int = 16.174	0.987	112.8
*L. tarentolae*	−3.431	y-int = 17.198	0.997	95.6
*L. infantum* + *L. tarentolae*	−3.359 −3.315	y-int = 15.505 y-int = 19.886	0.984 0.955	98.5 100.3
Q3 qPCR				
*L. infantum*	−2.988	y-int = 13.89	0.9997	116.1
*L. tarentolae*	−3.843	y-int = 7.841	0.9989	82.06
*L. infantum* + *L. tarentolae*	−3.448 −3.460	y-int = 9.492 y-int = 14.78	0.9967 0.9825	94.99 94.54
ddPCR				
*L. infantum*	0.9933	y-int = 7.262	0.996	–^*a*^
*L. tarentolae*	0.9468	y-int = 6.818	0.992	–
*L. infantum* + *L. tarentolae*	1.069 1.074	y-int = 7.611 y-int = 7.152	0.9999 0.9998	– –

^*a*^ –, not applicable.

**Fig 4:**
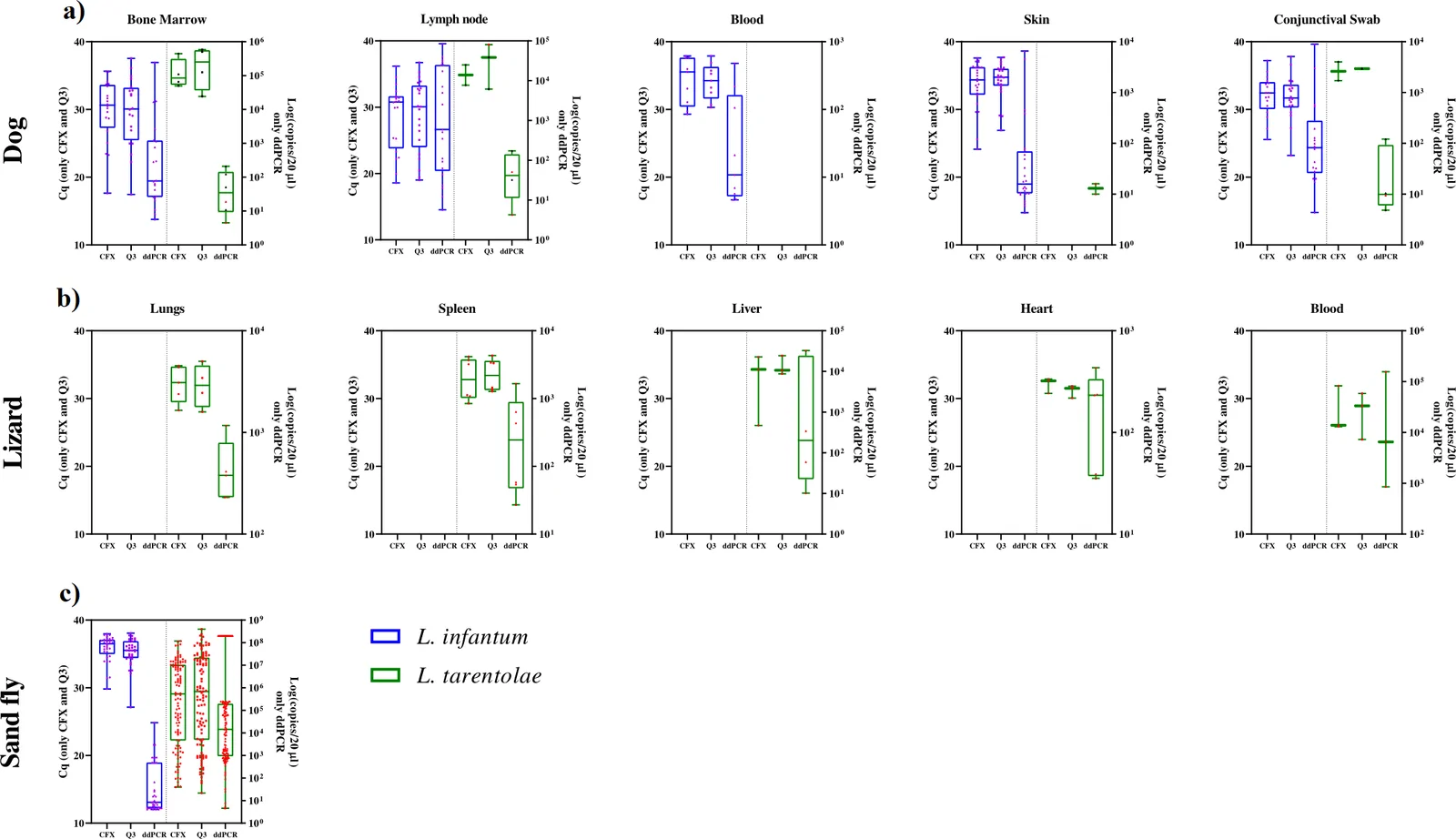
Boxplots of positive samples for *L. infantum* and *L. tarentolae* from different tissue sources and hosts (**a**, dog; **b**, lizard; **c**, sand fly) according to concentrations (ng/µL) and quantification cycle (Cq) values obtained after interpolation on the standard curve for each employed technique (CFX96 qPCR, Q3 qPCR, and ddPCR). Orange symbols and blue boxplots indicate *L. infantum*-positive samples; red symbols and green boxplots indicate *L. tarentolae*-positive samples; black symbols represent samples from dogs experimentally infected with *L. tarentolae*. Groups of samples with *n* ≤ 3 were not represented in the figure.

**Fig 5:**
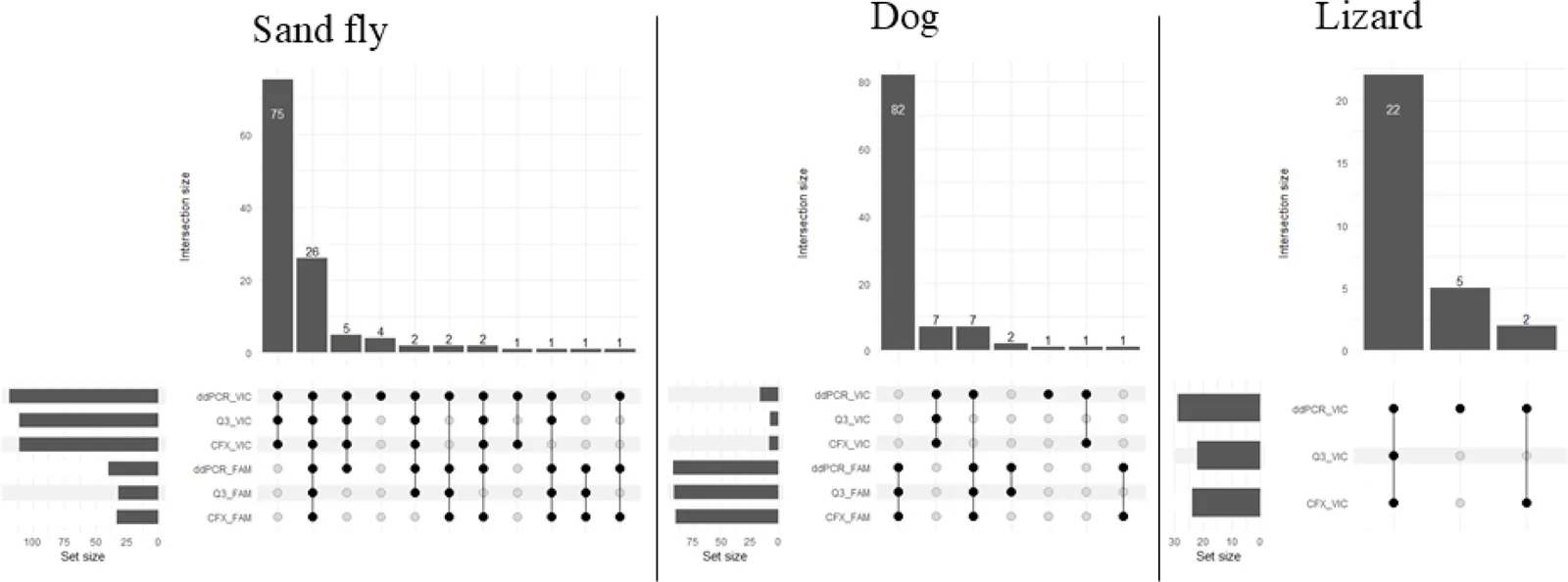
UpSet plots of positive samples according to platforms and fluorophores (FAM and VIC), divided by hosts (sand fly, dog and lizard).

Results of biological samples tested by the CFX96 and Q3 qPCRs were concordant in detecting the species of *Leishmania* (Table 1). In particular, samples from *Se. minuta* tested in the CFX96 and Q3 qPCRs scored positive for *L. infantum* (3.3%), *L. tarentolae* (67.5%), and both species (25.8%). The percentage of positivity (82.7%) of lizard samples for *L. tarentolae* was concordant between the CFX96 and Q3 qPCRs (Table 1, Fig. 4 and 5). Of the dogs naturally infected, 84.1% were positive for *L. infantum,* whereas 8.9% of those experimentally infected scored positive for *L. tarentolae*.

Overall, the mean Cq values (i.e., Cq values ranging from 27.93, corresponding to 5.76 × 10^−3^ ng/µL for a lizard blood sample positive for *L. tarentolae,* to 35.27, corresponding to 2.80 × 10^−7^ ng/µL for *Se*. *minuta* positive for *L. infantum*) in the CFX96 overlapped those of the Q3 qPCRs for most biological samples, with the exception of liver samples of lizard (mean Cq, 32.15 for CFX96 *vs* 34.72 for Q3) (Table 1).

**Fig 6:**
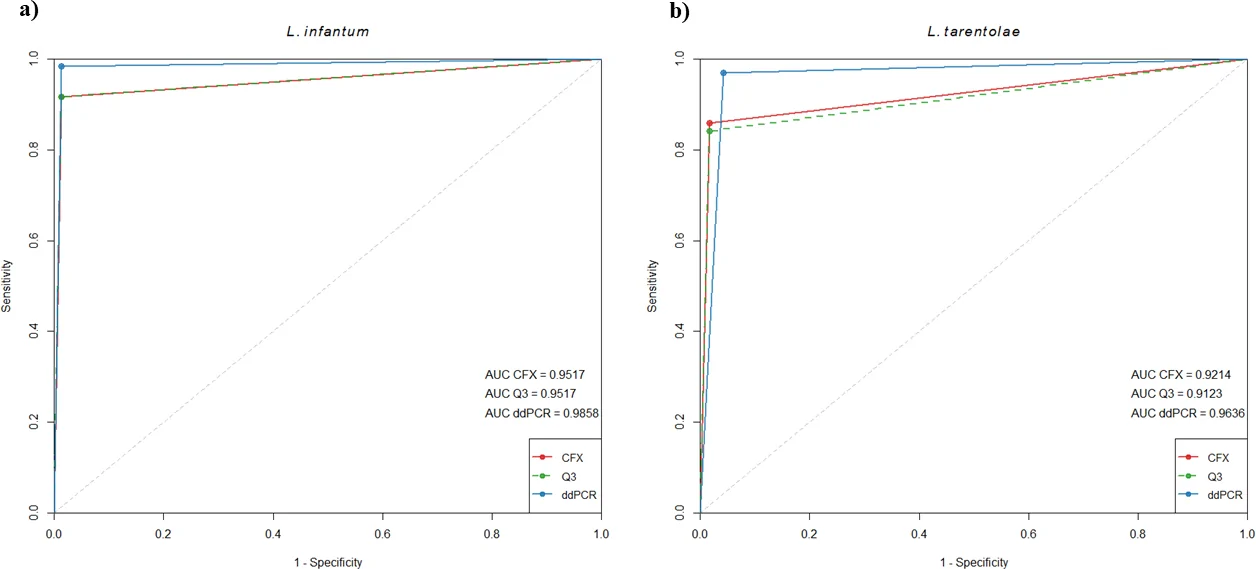
Receiver operating characteristic (ROC) curves obtained for each platform (CFX96 qPCR, Q3 qPCR, and ddPCR) according to *Leishmania* spp. (**a**, *L. infantum*; **b**, *L. tarentolae*).

All biological samples tested positive by qPCRs were also positive by ddPCR (Table 1). In addition, four *Se. minuta* (3.4%, copies/20 μL, 7.954 to 92.051, corresponding to 5.62 × 10^−7^ ng/µL to 7.47 × 10^−6^ ng/µL and Cq values of 39.04 to 35.46) and five lizard samples (17.2%, copies/20 μL, 7.201 to 53.48, corresponding to 5.06 × 10^−7^ ng/µL to 4.21 × 10^−6^ ng/µL and Cq values of 39.18 to 36.25) were positive for *L. tarentolae* exclusively in ddPCR (Fig. 4 and 5). Seven samples (i.e., two bone marrow, two lymph node, two skin, and one conjunctival swab sample) from dogs naturally infected by *L. infantum* were co-infected with *L. tarentolae* (copies/20 μL up to 50.839 for *L. tarentolae,* corresponding to 8.5 × 10^−6^ ng/µL and Cq of 36.33). A single conjunctival swab sample of a dog experimentally infected with *L. tarentolae* (4.83 copies/20 μL, corresponding to 9.50 × 10^−7^ ng/µL and Cq of 39.77) resulted positive in ddPCR (Fig. 4 and 5). The LOD recorded for biological samples by the Q3 overlapped those of the CFX96 qPCR, with the exception of two lizard tissues and one sand fly positive for *L. infantum* (Table 3). The LOD of serial dilutions of DNA from biological samples overlapped among the three devices, with the exception of some samples from dog tissues (e.g., conjunctival swab and lymph node) and lizard tissue (e.g., eggs and lungs) (Table 3). ROC analysis showed the highest AUC for ddPCR (0.9858 for *L. infantum*, 95% CI, 0.9717–0.9998; AUC, 0.9636 for *L. tarentolae,* 95% CI, 0.9284–0.9987) compared with that of the CFX96 qPCR (AUC, 0.9517; 95% CI 0.9045–0.9989 for *L. infantum*; AUC, 0.9214; 95% CI, 0.8453–0.9976 for *L. tarentolae*) and of Q3 qPCR (AUC, 0.9517; 95% CI, 0.9045–0.9989 for *L. infantum*; AUC, 0.9123 for *L. tarentolae*; 95% CI, 0.8273–0.9973) (Fig. 6).

**TABLE 3:** Limit of detection (LOD) calculated by testing serial dilutions of positive biological samples for *Leishmania* spp., expressed in quantification cycles (Cq) and copy number on 20 µL of reaction, according to CFX96 qPCR, Q3 qPCR, and ddPCR results^*a*^

CFX96 qPCR (Cq ± SD)						Q3 qPCR (Cq ± SD)					
Dog											
*Leishmania infantum*											
Serial dilutions	Bone marrow	Lymph node	Skin	Blood	Conjunctival swab	Bone marrow		Lymph node	Skin	Blood	Conjunctival swab
10^0^	19.5 ± 0.27	18.15 ± 0.05	25.30 ± 0.34	29.64 ± 0.12	25.63 ± 0.09	20.6 ± 0.09		20.1 ± 0.29	26.1 ± 1.13	30.4 ± 0.44	23.2 ± 0.07
10^−1^	22.52 ± 0.01	21.34 ± 0.29	28.3 ± 0.35	33.79 ± 0.83	28.61 ± 0.12	23.75 ± 0.21		23.5 ± 0.42	28.99 ± 0.43	33.3 ± 0.84	27.2 ± 0.20
10^−2^	25.45 ± 0.35	23.95 ± 0.07	31.30 ± 0.34	36.95 ± 0.21	31.61 ± 0.08	26.15 ± 0.07		27.2 ± 0.98	31.9 ± 0.07	36.8 ± 0.07	30.4 ± 0.35
10^−3^	28.56 ± 0.06	27.3 ± 0.28	34.27 ± 0.25	–^*b*^	34.74 ± 0.12	29.26 ± 0.04		30.31 ± 0.58	33.8 ± 0.14	–	34.2 ± 0.41
10^−4^	31.56 ± 0.24	30.34 ± 0.23	37.32 ± 0.49	–	37.89 ± 0.05	32.4 ± 0.56		32.95 ± 0.21	37.1 ± 0.35	–	37.3 ± 0.28
10^−5^	34.06 ± 0.01	33.83 ± 0.9	–	–	–	34.84 ± 0.19		36.4 ± 0.07	–	–	–
10^−6^	37.45 ± 0.12	37.12 ± 0.01	–	–	–	37.55 ± 0.21		–	–	–	–

*Leishmania tarentolae*											
Serial dilutions	Bone Marrow	Lymph node	Conjunctival swab	Bone Marrow				Lymph node	Conjunctival swab		
10^0^	33.49 ± 0.16	33.40 ± 0.14	37.34 ± 0.29	32.96 ± 0.27				33.12 ± 0.12	37.03 ± 0.48		
10^−1^	37.26 ± 0.34	36.45 ± 0.35		36.59 ± 0.40				36.51 ± 0.20			

Lizard											
*Leishmania tarentolae*											
Serial dilutions	Heart	Liver	Blood	Lung	Spleen	Eggs	Heart	Liver	Blood	Lung	Spleen
10^0^	31.45 ± 0.35	25.61 ± 0.56	28.11 ± 0.56	28.6 ± 0.282	31.21 ± 0.21	30.03 ± 0.27	30.08 ± 0.03	33.6 ± 0.23	29.1 ± 0.28	28.35 ± 0.21	30.79 ± 0.07
10^−1^	34.51 ± 0.282	28.61 ± 0.56	31.26 ± 0.33	31.6 ± 0.282	33.39 ± 0.21	33.51 ± 0.07	32.35 ± 0.21	36.8 ± 0.15	32.4 ± 0.02	31.45 ± 0.07	33.51 ± 0.25
10^−2^	37.51 ± 0.28	31.11 ± 0.08	34.53 ± 0.001	34.42 ± 0.28	35.85 ± 0.08	36.27 ± 0.06	35.61 ± 0.17	–	36.8 ± 1.7	35.16 ± 0.89	36.18 ± 0.16
10^−3^	–	34.51 ± 0.233	37.27 ± 0.59	37.82 ± 0.56	–	–	–	–	–		–
10^−4^	–	37.25 ± 0.15	–	–	–	–	–	–	–		–

Sand fly											
Serial dilutions	*L. tarentolae*	*L. infantum*	*L. infantum + L. tarentolae*		*L. tarentolae*			*L. infantum*	*L. infantum + L. tarentolae*		
10^−1^	18.3 ± 0.27	37.05 ± 0.06	36.5 ± 0.08	23.57 ± 0.10	15.83 ± 0.16			35.77 ± 0.06	33.72 ± 0.33	23.42 ± 0.31	
10^−2^	21.33 ± 0.29	–	–	26.48 ± 0.24	18.6 ± 0.33			–	36.5 ± 0.21	26.31 ± 0.21	
10^−3^	24.1 ± 0.014	–	–	29.01 ± 0.219	21.3 ± 0.42			–	–	30.1 ± 0.51	
10^−4^	27.74 ± 0.134	–	–	32.01 ± 0.176	24.53 ± 0.04			–	–	32.2 ± 0.34	
10^−5^	31.11 ± 0.17	–	–	35.96 ± 0.084	27.31 ± 0.16			–	–	34.9 ± 0.24	
10^−6^	33.97 ± 0.25	–	–	–	30.3 ± 0.28			–	–	–	
10^−7^	36.87 ± 0.11	–	–	–	33.57 ± 0.19			–	–	–	
10^−8^	–	–	–	–	36.78 ± 0.09			–	–	–	

ddPCR (copies/20 µl ± SD; Cq ± SD)											
Dog											
*L. infantum*						*L. tarentolae*					
Serial dilutions	Bone marrow	Lymph node	Skin	Blood	Conjunctival swab	Bone marrow		Lymph node	Conjunctival swab		
10^0^	2.45 x 10^5^ ± 507.58 (20.18 ± 0.04)	8.50 x 10^4^ ± 913 (21.88 ± 0.017)	6.56 x 10^3^ ± 16 (26.00 ± 0.05)	4.79 x 10^2^ ± 61.3 (30.21 ± 0.2)	8.98 x 10^3^ ± 260.2 (25.50 ± 0.06)	2.1 x 10^2^ ± 13.7 (34.26 ± 0.096)		1.72 x 10^2^ ± 16.3 (34.55 ± 0.16)	7.70 ± 1.21 (39.09 ± 0.253)		
10^−1^	3.48 x 10^4^ ± 408.3 (23.32 ± 0.02)	9.62 x 10^3^ ± 76 (25.39 ± 0.013)	1.83 x 10^3^ ± 11 (28.05 ± 0.01)	9.51 x 10^1^ ± 6.2 (32.81 ± 0.10)	1.22 x 10^3^ ± 98.1 (28.70 ± 0.13)	9.96 ± 1.22 (38.71 ± 0.196)		2.43 x 10^1^ ± 3.7 (37.41 ± 0.24)	–		
10^−2^	6.72 x 10^3^ ± 94 (25.96 ± 0.02)	1.08 x 10^3^ ± 57 (28.89 ± 0.074)	3.86 x 10^2^ ± 54 (30.55 ± 0.20)	1.14 x 10^1^ ± 0.35 (36.21 ± 0.05)	1.10 x 10^2^ ± 18.3 (32.56 ± 0.24)	–		3.71 ± 0.58 (40.15 ± 0.22)	–		
10^−3^	4.61 x 10^2^ ± 36 (30.27 ± 0.09)	2.15 x 10^2^ ± 28 (31.5 ± 0.212)	6.18 x 10^1^ ± 7 (33.50 ± 0.2)	-	5.42 ± 0.77 (37.41 ± 0.21)	–		–	–		
10^−4^	8.92 x 10^1^ ± 1.3 (32.91 ± 0.03)	2.61 x 10^1^ ± 4.9 (34.88 ± 0.211)	7.31 ± 0.65 (36.93 ± 0.17)	–	–	–		–	–		
10^−5^	2.11 x 10^1^ ± 0.1 (35.23 ± 0.02)	3.11 ± 0.15 (38.31 ± 0.070)	–	–	–	–		–	–		
10^−6^	5.29 ± 0.38 (37.45 ± 0.13)	–	–	–	–	–		-	-		

Lizard							Sand fly				
Serial dilutions	Heart	Liver	Blood	Lung	Spleen	Eggs					
*L. tarentolae*							*L. tarentolae*		*L. infantum*	*L. infantum + L. tarentolae*	
10^0^	4.32 x 10^2^ ± 9.24 (33.20 ± 0.03)	3.24 x 10^4^ ± 1036.8 (26.90 ± 0.051)	1.56 x 10^5^ ± 589.3 (24.60 ± 0.01)	1.17 x 10^3^ ± 93.5 (31.75 ± 0.12)	1.65 x 10^3^ ± 118.2 (31.24 ± 0.11)	1.23 x 10^3^ ± 57.3 (31.7 ± 0.08)	2.00 x 10^8^ ± 0 (14.15 ± 0)		5.29 ± 0.38 (37.45 ± 0.122)	2.56 x 10^1^ ± 1.74 (34.91 ± 0.095)	
10^−1^	3.09 x 10^1^ ± 0.28 (37.06 ± 0.02)	7.79 x 10^3^ ± 399.5 (28.98 ± 0.073)	8.85 x 10^2^ ± 154.1 (32.16 ± 0.25)	2.84 x 10^2^ ± 3.53 (33.82 ± 0.02)	1.77 x 10^2^ ± 4.24 (34.51 ± 0.05)	1.92 x 10^2^ ± 15.5 (34.39 ± 0.092)	5.25 x 10^7^ ± 24587.3 (16.1 ± 0.001)		–	3.51 ± 0.62 (38.11 ± 0.283)	
10^−2^	4.21 ± 0.015 (39.9 ± 0.001)	8.43 x 10^2^ ± 24.7 (32.23 ± 0.058)	7.56 x 10^1^ ± 5.2 (35.7 ± 0.119)	3.88 x 10^1^ ± 2.1 (36.7 ± 0.105)	2.08 x 10^1^ ± 0.9 (37.6 ± 0.062)	1.66 x 10^1^ ± 1.5 (37.96 ± 0.147)	3.57 x 10^6^ ± 6431.7 (20.03 ± 0.01)		–	–	
10^−3^	–	5.52 x 10^1^ ± 1.9 (36.21 ± 0.054)	8.23 ± 0.69 (39.03 ± 0.13)	3.45 ± 0.35 (40.26 ± 0.16)	-	4.65 ± 0.21 (39.83 ± 0.07)	2.19 x 10^5^ ± 1278 (24.11 ± 0.1)		–	–	
10^−4^	–	5.6 ± 0.3 (39.55 ± 0.102)	–	–	–	–	1.49 x 10^4^ ± 65 (28.03 ± 0.01)		–	–	
10^−5^	–	–	–	–	–	–	1.12 x 10^3^ ± 8.5 (31.81 ± 0.011)		–	–	
10^−6^	–	–	–	–	–	–	9.47 x 10^1^ ± 1.5 (35.42 ± 0.025)		–	–	
10^−7^	–	–	–	–	–	–	1.30 x 10^1^ ± 1.06 (38.32 ± 0.124)		–	–	

^*a*^ SD, Standard deviation.

^*b*^ –, not applicable.

## DISCUSSION

The duplex qPCR assay for the detection of *L. infantum* and *L. tarentolae* was efficient when applied in all three devices used. In particular, the low amount of DNA detected by all devices in testing serial dilutions of DNA from *Leishmania* cultures and from biological samples indicates the high analytical and clinical sensitivity of the assay. The results were overall consistent between qPCR’s devices, with a minor difference in the Cq values for *L. tarentolae* DNA in liver samples from lizard (32.15 for CFX96 *vs* 34.72 for Q3). The latter could be the result of genomic degradation or depletion. Conversely, the higher number of positive samples in ddPCR *vs* qPCR (6.8% of samples) suggests higher sensitivity of the ddPCR, as also supported by the highest AUC value (up to 0.9858) compared with qPCRs (AUC, 0.9517 for CFX96 and Q3 qPCRs). This difference could be due to the fact that ddPCR technology is less susceptible to PCR inhibitors when testing clinical and field samples (21, 24, 25). Indeed, the technology involved in ddPCR device (i.e., partitioning the reaction into picoliter droplets) increases sensitivity in target detection while reducing interference from inhibitors and determines high-confident measurement of the targeted molecules without the need for calibration curves (26, 27). However, the negative results obtained by CFX96 and Q3 qPCR devices could be due to the corresponding high Cq values (i.e., 36.25 to 39.95) in qPCRs assays. Indeed, the main limitation of the qPCR technologies was observed when testing field samples with high Cq values close to the upper limit of the threshold cycle (28). When some field samples (e.g., lizard samples) were tested by qPCRs, the upper limits of Cq values were likely due to minimal pathogen load and/or PCR inhibitors (i.e., lungs, ddPCR copies/20 μL, 4.65 ± 0.21, corresponding to a Cq 39.83 ± 0.071; eggs, copies/20 μL 5.6 ± 0.3, corresponding to a Cq value of 39.55 ± 0.102). On the other hand, although ddPCR showed advantages over qPCRs (e.g., absolute quantification without the need for a calibration curve), it cannot quantify the DNA amounts when testing samples with high concentrations (e.g., in some sand fly midgut samples with 2 × 10^8^ copies/20 μL of *L. tarentolae* DNA), resulting in saturation of positive droplets and impairing DNA quantification (29). In addition, the procedures with ddPCR are more time-consuming and expensive. Considering the overlapping results by testing samples with CFX96 and Q3 qPCRs, the latter could be used as a point-of-care diagnostic device, even in the absence of clinical laboratory facilities and/or skilled personnel. In addition, another important advantage of the Q3 qPCR was the lower amount of reagent requirement for each reaction (final volume, 5 μL for Q3 *vs* 20 μL for CFX96) in samples screening (19). Indeed, though several assays have been standardized for the traditional qPCR (17, 28), this technique requires standard laboratory infrastructure, which may impair its use, particularly in resource-limited settings (19).

Regarding the biological samples, the finding of both *Leishmania* spp. in *Se*. *minuta* provided further evidence of the involvement of this vector in the circulation of *L. infantum* and *L. tarentolae* in endemic areas (9, 10, 21, 30), which ultimately may promote the occurrence of genetic recombination events (31, 32). Overall, the presence of hybrids represents a potential challenge to medical and veterinary practitioners by increasing diagnostic inaccuracies due to cross-reactivity in the analyses used. For example, the development of hybrids may contribute to the appearance of peculiar clinical presentations of human leishmaniasis (33), as well as new tropism in tissue infection within the same or even different host species (32). This could explain the detection of *L. tarentolae* in some tissues (i.e., bone marrow and lymph nodes) of naturally infected dogs for *L. infantum*, as previously supported by *in vivo* and *in vitro* experiments (14, 15).

In particular, the finding of *L. tarentolae* DNA at high copy number in bone marrow (i.e., copies/20 μL 18.75) and lymph node (i.e., copies/20 μL 50.84) samples supports previous observations about the tissue tropism of this species of *Leishmania,* as well as the hypothesis that this species may have the capacity to visceralize in naturally infected dogs (14, 15). Furthermore, the detection of *L. tarentolae* DNA in both skin (i.e., copies/20 μL 16) and conjunctival swab (i.e., copies/20 μL 9.37) samples suggests that these tissues may serve as less invasive alternatives for the molecular detection of *L. tarentolae* in dogs.

In conclusion, in spite of the limitations of kDNA as a marker for quantitative molecular analysis due to its variability in copy number among *Leishmania* species, the assay developed in this study may help improve current knowledge on the distribution of *L. tarentolae*, other than *L. infantum*, and better understand the role of lizards, *Se. minuta,* and dogs as potential new hosts/reservoirs or vectors for these *Leishmania* spp. Furthermore, the assay implemented on both the CFX96 and Q3 qPCR devices is suitable for large-scale sample testing and facilitates use in specialized and non-specialized laboratories, respectively. Conversely, the assay applied on ddPCR may be advantageous for detecting target DNA in low quantities or when inhibitors (i.e., blood, eggs, and lungs) are present.
